# HCV and HBV prevalence based on home blood self-sampling and screening history in the general population in 2016: contribution to the new French screening strategy

**DOI:** 10.1186/s12879-019-4493-2

**Published:** 2019-10-28

**Authors:** Cécile Brouard, Leïla Saboni, Arnaud Gautier, Stéphane Chevaliez, Delphine Rahib, Jean-Baptiste Richard, Francis Barin, Christine Larsen, Cécile Sommen, Josiane Pillonel, Elisabeth Delarocque-Astagneau, Nathalie Lydié, Florence Lot, the 2016 Health Barometer Group

**Affiliations:** 1 Santé publique France, the national public health agency, HIV, Hepatitis B/C and STI Unit, Saint-Maurice, France; 2 Santé publique France, the national public health agency, Surveys Unit, Saint-Maurice, France; 3 National Reference Centre for Viral Hepatitis B, C and Delta, Department of Virology, Henri Mondor University Hospital, Créteil, France; 4 INSERM U955, Paris-Est University, Créteil, France; 5 Santé publique France, the national public health agency, Sexual Health Unit, Saint-Maurice, France; 6 National Reference Centre for HIV, Department of Virology, Bretonneau University Hospital, Tours, France; 7 INSERM U1259, François-Rabelais University, Tours, France; 8 Santé publique France, the national public health agency, Biostatistics Unit, Saint-Maurice, France; 9 INSERM 1181, Biostatistics, Biomathematics, Pharmacoepidemiology, and Infectious Diseases (B2PHI), Paris, France; 10 Pasteur Institute, B2PHI, Paris, France; 11 Versailles Saint-Quentin University UMR 1181, B2PHI, Montigny-le-Bretonneux, France

**Keywords:** Hepatitis C, Hepatitis B, Prevalence, Screening, France

## Abstract

**Background:**

The advent of effective direct-acting antivirals (DAAs), has prompted an assessment of the French Hepatitis C virus (HCV) screening strategy, which historically targeted high-risk groups. One of the options put forward is the implementation of combined (i.e., simultaneous) HCV, Hepatitis B virus (HBV) and HIV screening for all adults at least once during their lifetime (“universal combined screening”). However, recent national survey-based data are lacking to guide decision-making regarding which new strategy to implement. Accordingly, we aimed to provide updated data for both chronic hepatitis C (CHC) and B (CHB) prevalence and for HCV and HBV screening history, using data from the BaroTest and 2016 Health Barometer (2016-HB) studies, respectively.

**Methods:**

2016-HB was a national cross-sectional phone based health survey conducted in 2016 among 20,032 randomly selected individuals from the general population in mainland France. BaroTest was a virological sub-study nested in 2016-HB. Data collected for BaroTest were based on home blood self-sampling on dried blood spots (DBS).

**Results:**

From 6945 analyzed DBS, chronic hepatitis C (CHC) and B (CHB) prevalence was estimated at 0.30% (95% Confidence Interval (CI): 0.13-0.70) and 0.30% (95% CI: 0.13-0.70), respectively. The proportion of individuals aware of their status was estimated at 80.6% (95% CI: 44.2-95.6) for CHC and 17.5% (95% CI: 4.9-46.4) for CHB. Universal combined screening would involve testing between 32.6 and 85.3% of 15-75 year olds according to whether we consider only individuals not previously tested for any of the three viruses, or also those already tested for one or two of the viruses.

**Conclusions:**

Our data are essential to guide decision-making regarding which new HCV screening recommendation to implement in France. They also highlight that efforts are still needed to achieve the WHO’s targets for eliminating these diseases. Home blood self-sampling may prove to be a useful tool for screening and epidemiological studies.

## Background

Chronic hepatitis C (CHC), chronic hepatitis B (CHB) and HIV infection are major public health issues worldwide, affecting, respectively, 71, 257 and 37 million people [1, 2]. These infections have some similarities in terms of key populations (e.g. people who inject drugs, migrants, men who have sex with men (MSM)), and epidemiological features. In 2014, the arrival of direct-acting antivirals (DAAs) revolutionized the treatment of CHC, leading to viral elimination in 90-95% of patients. Existing antiviral treatments control CHB and HIV in the majority of treated patients. Therapies for these three diseases are effective at reducing the risk of complications and mortality, and help to prevent viral transmission. This raises hope that the World Health Organization’s (WHO) target to end the CHC, CHB and HIV epidemics by 2030 is possible [2, 3]. However, an essential step in this process is to significantly increase the proportion of diagnosed infections to 90% (by 2020 for HIV and by 2030 for CHC and CHB).

France is a low-endemic country for these three infections. Prevalence in the general adult population is estimated at 0.36% for HIV (for 2016), 0.42% for CHC (for 2011) and 0.65% for CHB (for 2004) [4–6]. Despite these low values, much still needs to be done to achieve the WHO targets, in particular for CHC and CHB. More specifically, the proportion of French people aware of their infection is estimated at 86% for HIV (for 2016), but only at 57% for CHC (for 2004) and 45% for CHB (for 2004) [6–8]. Nonetheless, screening activity is quite high, with the number of tests performed in public and private laboratories equaling 81 (HIV), 62 (Hepatitis C virus (HCV)) and 65 (Hepatitis B virus (HBV)) per 1000 inhabitants in 2016 [9, 10]. Moreover, testing tools have diversified in recent years with the development of rapid diagnosis tests for all three diseases and HIV self-tests [8, 11].

For a long time in France, HIV screening strategy only targeted key populations (“risk-based strategy”). In 2009 and again in 2017, the French National Authority for Health (HAS) recommended complementing HIV risk-based testing strategy with screening of all individuals aged 15 years and over at least once during their lifetime, irrespective of their risk exposure (“universal screening”) [11]. These recommendations were based on cost-effectiveness analyses [12].

While very recent screening recommendations (2017) [11] and prevalence and diagnosis data (2016) [6] exist for HIV in France, the situation is, unfortunately, very different for HCV and HBV. Indeed, current official recommendations - which promote risk-based strategies - have not been reassessed for nearly 20 years [13, 14]. With regard to estimations of CHC and CHB prevalence and the proportion of individuals aware of their chronic infection in the general population, the most recent survey-based figures date back more than 15 years and are based on a large national prevalence survey conducted in 2004, which could not been renewed due to its prohibitive cost [5]. Since then, CHC and CHB prevalences have been estimated using modeling studies [4, 15–17], all largely based on data from the 2004 survey [5]. Given the evolution of the epidemiological context since then, in particular regarding HCV, these model-based estimates can no longer be deemed accurate and new survey-based prevalence data are essential.

At the request of the French Ministry of Health, and in the context of the provision of free DAAs being expanded to all patients with CHC since 2016 [18], the HAS is currently re-evaluating the HCV screening strategy. The main option being considered is the implementation of combined (i.e., simultaneous) HCV, HBV and HIV screening for all adults (i.e. “universal combined screening”) at least once during their lifetime as a complementary measure to existing risk-based testing [19]. This option has been proposed in several expert reports in recent years [8, 20] and has been shown in models to be cost-effective in France in the context of universal treatment [21]. However, these models also relied on data from the 2004 prevalence survey [5].

In 2016, BaroTest, an innovative virological study investigating HCV, HBV and HIV, based on home blood self-sampling and nested in a large national health survey (2016 Health Barometer) conducted in the French general population, finally made it possible to generate new estimates for CHC and CHB prevalences.

The aim of this paper is to provide estimates for current HCV and HBV prevalences in mainland France, especially given the context of the ongoing reassessment of current HCV screening recommendations [19]. More specifically, it aims to provide 2016 estimates in the general population for the following: 1) the prevalences of CHC and CHB; 2) the proportions of persons with CHC or CHB aware of their infection; 3) the proportions of people with a lifetime history of HCV or HBV testing and their characteristics; 4) the number of people that would be tested if the option of a universal combined screening strategy were implemented.

## Methods

The protocol of the BaroTest survey has recently been published [22].

### Study design

BaroTest participants were recruited using the 2016 Health Barometer (2016-HB), which was a national cross-sectional telephone survey on health behaviors and perceptions, conducted during the first semester of 2016 among a representative sample of the general population aged 15 to 75 years, able to speak French and living in mainland France (20,032 participants: 15,216 from the national sample, 4816 from regional subsamples). The 2016-HB sampling method was based on a random generation of landline and cellular phone numbers, then on a random selection of one individual among eligible household members [23]. During the 40-min-long phone interview, in addition to socio-demographical characteristics, data related to HCV, HBV and HIV were collected as follows: history of testing during lifetime (“Have you ever been tested for hepatitis C / hepatitis B / HIV?”), result of the most recent test(s) (“Positive/negative/I don’t know” for HCV and HIV; “I do not have hepatitis B/I had hepatitis B, but I am cured/I have hepatitis B/ I don’t know” for HBV), history of HBV vaccination and risk exposure factors (e.g. blood transfusion, intravenous or nasal drug use, medical care or prolonged stay in Africa, Asia or Middle East, household (i.e. living under the same roof) or sexual contact with an HBV-infected person, tattooing or piercing).

At the end of the interview, eligible participants for the BaroTest sub-study, i.e. persons aged 18 to 75 years, having health insurance coverage and not under guardianship, were invited to benefit from free HCV, HBV and HIV screening by home blood self-sampling. A self-sampling kit was sent by postal mail to the homes of those who agreed to participate. They performed self-administered fingerprick blood sampling on dried blood spots (DBS) and then sent these with a signed informed consent form (including the name and address of their general practitioner (GP)) by postal mail to the National Reference Centre (NRC) in charge of virological analyses (Additional file 1: Figure S1).

### Laboratory testing

After elution of the DBS, detection of total HCV antibodies and HBsAg were performed by means of automated enzyme immunoassays according to the manufacturer’s instructions (aHCV Vitros ECi, Ortho-Clinical Diagnostics, Raritan, New Jersey, USA; VIDAS HBsAg Ultra, BioMerieux, France, respectively). In anti-HCV-positive samples, a real-time PCR based method was used to detect HCV RNA (Abbott RealTime HCV assay, Abbott Molecular, Des Plaines Illinois, USA) [24, 25].

In case of negative results for HBsAg, and for anti-HCV and anti-HIV antibodies, the NRC informed the participant and the GP by postal mail. If at least one test was positive, the NRC sent the results of all the tests to the GP with a letter informing him/her that the participant had been invited to contact him/her to obtain the results (Additional file 1: Figure S1).

### Data analysis

#### Outcome

CHC was defined by the detection of HCV RNA, and CHB by the detection of HBsAg.

The prevalences of CHC and CHB were defined as the proportions of persons testing positive among the tested population and extrapolated to all 18-75 year olds in the general population in mainland France, using the 2016 population estimate from the French institute for statistics and economic studies (Insee). For each of these two diseases, the proportion of infected persons aware of their infection was defined as the proportion of people among those testing positive in the BaroTest study, who reported during the 2016-HB telephone interview that they had been previously screened for the disease and who replied “positive” when asked about the result of their most recent HCV test and/or replied “I have hepatitis B” when asked about HBV. The proportion of the general population that would need to be tested with the universal combined screening option was estimated according two scenarios: i) only people reporting that they had not previously been tested for any of the three virus (minimal estimate); ii) the population from the first scenario plus people reporting to have previously been tested for one or two of the three viruses (maximal estimate). Subsequently, the numbers of people that would need to be tested were estimated by applying these proportions to the total number of persons aged 15 to 75 years living in mainland France in 2016 (Insee).

#### Sample size

Based on a prevalence of 0.65% for HBsAg (the highest expected prevalence) and an accuracy of 0.22%, we estimated a minimum study sample size of 5,000 persons.

#### Statistical analysis

2016-HB data were weighted to take into account the probability of inclusion and were adjusted for sociodemographic data using the 2014 Insee Labour Force Survey (HB weight indicated as*“wHB”*) [23].

For the BaroTest sample, additional weights were constructed. The first was to take into account any differential participation rate according to gender, age, country of birth, region, educational level, household monthly income and level of urbanization of the place of residence. To do this, the quantile score method was used. Following a logistic regression estimating the response probability, the sample was divided into ten groups of equal size according to the response probability predicted by the model. In each group, the inverse of the observed response rate within each group was used as a corrective factor for non-response [26–29]. A second additional weight was constructed to keep the socio-demographical structure of the BaroTest sample similar to that of the national population using Insee data. The final BaroTest weight is indicated as *“wBT”*.

For the analyses, individuals were grouped according to the level of endemicity of HCV and HBV in their country of birth [30]. Multivariate Poisson regression models were used to identify factors independently associated with HCV or HBV lifetime screening history. Analyses were performed with Stata 13 software (Stata-Corp. USA).

## Results

### Participation and population characteristics

**Fig. 1:**
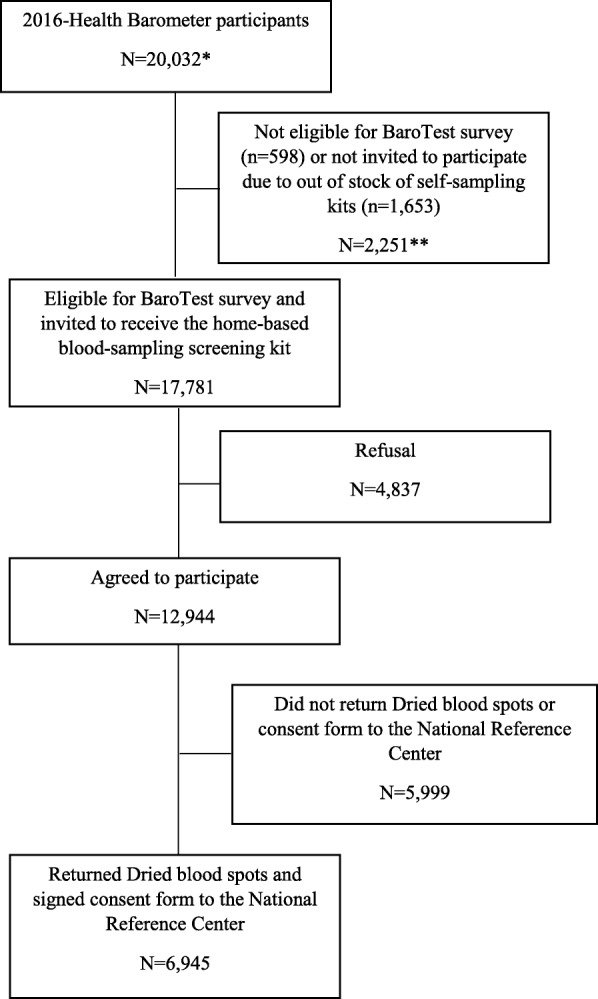
*15,216 participants were recruited from the national sample and 4,816 from regional subsamples. ** 499 persons were not eligible because they were aged of 15-17 years and 99 because they did not have health insurance coverage or because they were under guardianship. One thousand six hundred fifty-three persons were eligible to BaroTest but were not invited to participate to BaroTest due to out of stock of self-sampling kits

Of the 20,032 individuals included in the 2016-HB, 17,781 were eligible for BaroTest and were invited to receive the home-sampling testing kit (Fig. 1). Of the 12,944 (72.8%) who agreed to participate, 6,945 persons (53.7%) returned the DBS with the signed informed consent form to the NRC, representing 39.1% of all those initially invited to participate.

The characteristics of BaroTest participants and non-participants and 2016-HB participants are presented in Tables 1 and 2. Before BaroTest weighting, BaroTest participants (column 3) differed significantly from non-participants (column 4) for all socio-demographical characteristics, except gender. With regard to HCV and HBV risk exposure, BaroTest participants did not differ from non-participants, except for exposures related to country of birth (Table 2). Furthermore, BaroTest participants reported HBV (*p* = 0.03) or HIV (*p* < 10^− 3^) screening and vaccination against HBV (*p* < 10^− 3^) more frequently than non-participants.

**Table 1:** Socio-demographical characteristics in BaroTest and 2016-Health Barometer samples, mainland France, 2016

Characteristics		BaroTest (eligible, n = 17,781) 18-75 years old				2016-HB (n = 20,032) 15-75 years old
		Participants (n = 6,945)			Non participants (n = 10,836)	
		*wBT %* ^1^	*wHB* %^a^		*wHB* %^a^	*wHB* %^a^
Gender	Men	48.7	47.7		49.3	48.8
	Women	51.3	52.3		50.7	51.2
Age (in years)	18-30	22.1	**19.0**	***	**23.9**	25.4
	31-45	27.6	**26.3**		**27.3**	26.3
	46-60	29.1	**30.7**		**28.4**	27.9
	61-75	21.2	**24.0**		**20.4**	20.4
Educational level	< Secondary school certificate	49.7	**46.3**	***	**53.0**	52.1
	Secondary school certificate	20.0	**20.4**		**19.4**	19.2
	Higher education qualification	29.8	**33.1**		**27.3**	28.5
	Not specified	0.5	**0.2**		**0.3**	0.2
Place of residence in France	Ile-de-France (Paris region)	19.0	**15.6**	***	**18.7**	19.0
	North-West	19.9	**22.3**		**20.4**	19.9
	North-East	22.5	**22.7**		**23.7**	22.5
	South-East	20.5	**21.1**		**19.2**	20.5
	South-West	18.1	**18.3**		**18.0**	18.1
Level of urbanization	Rural	23.5	**27.3**	***	**22.1**	23.6
	< 20,000 inhabitants	16.6	**17.9**		**16.2**	16.7
	20,000 – 99,999 inhabitants	12.3	**11.7**		**13.1**	12.3
	≥ 100,000 inhabitants	31.2	**29.9**		**32.5**	31.1
	Paris urban area	16.4	**13.2**		**16.1**	16.3
Household monthly income	1st tercile (low)	34.5	**27.4**	***	**37.9**	34.9
	2nd tercile	31.1	**32.9**		**29.3**	30.2
	3rd tercile (high)	31.5	**37.8**		**27.9**	30.2
	Not specified	2.9	**1.9**		**4.9**	4.7
Place of birth	Mainland France	84.8	**90.6**	***	**85.9**	87.4
	French Overseas administrative areas	0.9	**0.9**		**1.2**	1.1
	Europe	4.4	**2.9**		**3.6**	3.4
	Maghreb	5.2	**3.0**		**4.7**	4.0
	Sub-Saharan Africa	2.1	**1.5**		**3.0**	2.7
	Other countries	2.6	**1.1**		**1.6**	1.4
Health Insurance coverage	General Health Insurance	91.6	**93.9**	***	**90.0**	90.0
	Health Insurance for low-income persons^b^	6.0	**4.5**		**7.6**	6.6
	Other^c^	2.4	**1.6**		**2.4**	3.4

*** *P*-value < 0.001 between BaroTest participants and non-participants wHB distributions using Chi-2 test

^a^*wHB %* Health Barometer weighted percentage, *wBT %* BaroTest weighted percentage

^b^Includes Complementary Universal Health Insurance (CMU, which is free insurance for low-income persons) and State Medical Assistance (AME, which is free insurance for low-income irregular migrants)

^c^Includes “No health coverage”, “Yes, but did not know which one” and “Not specified”

The distributions are significantly different for numbers in bold

**Table 2:** Risk exposure factors and prevention practices regarding HBV, HCV and HIV in BaroTest and 2016-Health Barometer samples, mainland France, 2016

Characteristics		BaroTest (eligible, *n* = 17,781)				2016-HB (*n* = 20,032)
		Participants (*n* = 6,945)			Non participants (*n* = 10,836)	
		*wBT %* ^a^	*wHB* %^a^		*wHB* %^a^	*wHB* %^a^
HCV endemicity in country of birth^b^	Low	91.1	**94.8**	***	**91.4**	92.4
	Intermediate or high	8.9	**5.2**		**8.6**	7.6
HBV endemicity in country of birth^c^	Low	86.6	**91.8**	***	**86.8**	88.4
	Intermediate	10.2	**6.2**		**9.7**	8.4
	High	3.2	**2.0**		**3.5**	3.2
Healthcare or stays ≥3 months in Africa, Asia or Middle East	Yes	12.6	**11.0**	*	**12.5**	11.9
	No	87.4	**89.0**		**87.5**	88.1
Blood transfusion before 1992	Yes	6.3	6.8		6.2	6.0
	No	92.7	92.4		92.7	93.0
	Not specified	1.0	0.8		1.1	1.0
Drug use during lifetime	Yes, intravenous use with or without nasal use	0.6	0.5		0.4	0.4
	Yes, nasal use only	5.9	5.2		4.7	4.8
	No	93.5	94.3		94.9	94.8
Lived with or had sexual intercourse with an HBV-infected person	Yes	4.3	3.9		3.4	3.6
	No	95.3	95.8		96.2	96.0
	Not specified	0.4	0.3		0.4	0.4
Sex with other men during lifetime^d^	Yes	3.8	3.7		3.9	3.7
	No	96.1	96.2		95.7	96.0
	Not specified	0.1	0.1		0.4	0.3
Sexually transmitted infection(s) in the previous 12 months^e^	Yes	1.3	1.1		1.0	1.1
	No	98.7	98.9		99.0	98.9
Tattooing or piercing without single-use materials	Yes	2.1	2.0		2.1	2.0
	No	97.9	98.0		97.9	98.0
HCV screening during lifetime	Yes	19.9	19.2		19.8	19.2
	No	76.2	77.0		76.4	76.9
	Not specified	3.9	3.8		3.8	3.9
HBV screening during lifetime	Yes	37.4	**37.1**	*	**36.5**	35.6
	No	59.2	**59.5**		**60.9**	61.4
	Not specified	3.4	**3.4**		**2.6**	3.0
HIV screening during lifetime	Yes	60.3	**60.0**	***	**56.7**	57.8
	No	39.4	**39.7**		**43.0**	41.9
	Not specified	0.3	**0.3**		**0.3**	0.3
HBV vaccination	Yes	47.8	**47.0**	***	**44.7**	46.1
	No	45.9	**47.3**		**48.0**	46.6
	Not specified	6.3	**5.7**		**7.3**	7.3

* *P*-value < 0.05, ** *P* value < 0.01, *** *P* value < 0.001 between BaroTest participants and non-participants wHB distributions using Chi-2 test

^a^wHB %: Health Barometer weighted percentage; wBT %: BaroTest weighted percentage

^b^Countries with a low level of HCV endemicity: Europe, America, Caribbean; countries with an intermediate or high level of endemicity: Africa, Middle-East, Indian subcontinent, Asia, Pacific Islands

^c^Countries with a low level of HBV endemicity: Northern and Western Europe, North America, Pacific Islands; countries with an intermediate level of endemicity: French Overseasadministrative areas, Eastern and Southern Europe, North Africa, Middle-East, Indian subcontinent, South America; countries with a high level of endemicity: Sub-Saharan Africa, Asia

^d^Among men who reported having had sexual relations in their lifetime (with men or women)

^e^Among individuals who reported having already had sexual relations in their lifetime. Mycosis are excluded

The distributions are significantly different for numbers in bold

After BaroTest weighting, the distributions of the main socio-demographical characteristics of BaroTest participants (column 2) were similar to those in the national population.

With regard to risk exposure factors, 6.3% of BaroTest participants reported a blood transfusion before 1992, 0.6% intravenous (IV) drug use in their lifetime, 12.6% health care or a prolonged stay in Africa, Asia or the Middle East, 4.3% household or sexual contact with an HBV-infected person, 2.1% a tattoo or a piercing made without single-use materials, and 1.3% a sexually transmitted infection (STI, excluding mycosis) in the previous 12 months. Finally, 3.8% of male participants declared having sex with men during their lifetime.

### CHC and CHB prevalence estimates

HCV RNA was detected in 11 individuals. CHC prevalence in the general population aged 18-75 living in mainland France was estimated at 0.30% (95% Confidence interval (CI): 0.13-0.70), corresponding to 133,466 individuals (95% CI: 56,880-312,616). The prevalence did not significantly differ between men (0.34%) and women (0.26%) (Table 3). CHC prevalence was significantly higher in persons: i) 46-75 years old (0.51%) than those 18-45 years old (0.08%, *p* < 0.05), ii) with an educational level lower than secondary school certificate (0.52%) than those with a higher diploma (0.08%, *p* < 10^− 2^) and iii) with a household monthly income in the lowest tercile (0.74%) than those living in a household with a monthly income in the 2nd or the 3rd terciles (0.07%, *p* < 10^− 3^). With regard to HCV risk exposure factors, CHC prevalence reached 12.1% among those who reported IV drug use in their lifetime (vs. 0.24% for those reporting no drug use, *p* < 10^− 3^) and was significantly higher in those with a tattoo or piercing not made with single-use materials than in others (2.55% vs. 0.25%, *p* < 10^− 2^). CHC prevalence was not significantly higher in persons with a history of blood transfusion before 1992 (1.12%) than in those without such a history (0.24%).

**Table 3:** Estimated prevalence of CHC and CHB according to socio-demographical characteristics and risk exposure factors in the general population aged 18-75 years, BaroTest, mainland France, 2016

Characteristics		Chronic hepatitis C^a^ (n = 6,931)			Chronic hepatitis B^b^ (n = 6,945)		
		*wBT%*	95%CI	p	*wBT%*	95%CI	p
**Total**		**0.30**	**0.13-0.70**		**0.30**	**0.13-0.70**	
Gender	Men	0.34	0.14-0.84	NS	0.28	0.12-0.63	NS
	Women	0.26	0.05-1.21		0.32	0.08-1.28	
Age (in years)	18-45	**0.08**	**0.01-0.45**	**< 0.05**	**0.10**	**0.03-0.29**	**< 0.05**
	46-75	**0.51**	**0.20-1.32**		**0.51**	**0.19-1.34**	
Educational level	< secondary school certificate	**0.52**	**0.19-1.37**	**< 10** ^**−2**^	0.18	0.06-0.53	NS
	≥ secondary school certificate	**0.08**	**0.03-0.22**		0.19	0.10-0.38	
Household monthly income	1st tercile (low)	**0.74**	**0.27-1.96**	**< 10** ^**−3**^	**0.63**	**0.20-1.96**	**< 0.05**
	2nd/3rd tercile	**0.07**	**0.03-019**		**0.14**	**0.07-0.27**	
Place of residence	Ile-de-France (Paris region)	0.31	0.05-1.79	NS	0.16	0.04-0.60	NS
	Other regions	0.30	0.11-0.78		0.34	0.13-0.84	
Place of birth	Mainland France	0.35	0.15-0.82		**0.14**	**0.08-0.26**	**< 10** ^**−3**^
	French Overseas administrative areas	0			**0**		
	Europe	0			**0.96**	**0.13-6.48**	
	Maghreb	0			**0**		
	Sub-Saharan Africa	0			**5.81**	**0.95-28.5**	
	Other countries	0			**0.73**	**0.10-5.10**	
Health Insurance coverage for low-income persons^c^	Yes	0.66	0.09-4.5	NS	**1.98**	**0.43-8.59**	**< 10** ^**−3**^
	No	0.27	0.10-0.69		**0.16**	**0.09-0.28**	
Drug use during lifetime	Yes, intravenous use with or without nasal use	**12.1**	**2.93-38.4**	**< 10** ^**−3**^	0		NS
	Yes, nasal use only	**0**			0.24	0.03-1.71	
	No	**0.24**	**0.09-0.67**		0.31	0.13-0.74	
Blood transfusion before 1992	Yes	1.12	0.24-5.03	NS			NS
	No	0.24	0.09-0.67				
Healthcare or stays ≥3 months in Africa, Asia or Middle East	Yes	0.33	0.06-1.85	NS	0.95	0.14-6.01	NS
	No	0.29	0.11-0.76		0.21	0.11-0.40	
Tattooing or piercing without single-use materials	Yes	**2.55**	**0.36-15.9**	**< 10** ^**−2**^	0.9	0.10-6.17	NS
	No	**0.25**	**0.10-0.64**		0.29	0.12-0.70	
Lived with or sexual intercourse with an HBV infected person	Yes			NS	0.68	0.20-2.34	NS
	No				0.28	0.11-0.72	
Sex with other men during lifetime^d^	Yes	0.31	0.04-2.19	NS	**3.39**	**0.84-12.7**	**< 10** ^**−3**^
	No	0.34	0.14-0.87		**0.16**	**0.06-0.39**	
Sexually transmitted infection(s) in the previous 12 months^e^	Yes	0.46	0.06-3.22	NS	1.16	0.16-7.82	NS
	No	0.3	0.13-0.72		0.30	0.12-0.72	
HBV vaccination	Yes	0.17	0.05-0.58	NS	0.36	0.09-1.39	NS
	No	0.47	0.16-1.38		0.29	0.13-0.63	

*wBT%* BaroTest weighted percentage, *aPR* adjusted prevalence ratio, *95% CI* 95% confidence interval, *NS* Not significant

^a^Defined as positive HCV RNA

^b^Defined as positive HBs Ag

^c^Includes Complementary Universal Health Insurance (CMU, which is free insurance for low-income persons) and State Medical Assistance (AME, which is free insurance for low-income irregular migrants)

^d^Among men who reported having had sexual relations in their lifetime (with men or women)

^e^Among individuals who have already had sexual relations. Mycosis are excluded

The distributions are significantly different for numbers in bold

Among persons with CHC, 80.6% (95% CI: 44.2-95.6) were estimated to be aware of their infection, corresponding to 107,574 (95% CI: 58,992-127,594) people aged 18-75 years in the general population in mainland France.

Among the 6,945 persons tested for HBsAg, 18 were positive. CHB prevalence in the general population aged 18-75 years living in mainland France was estimated at 0.30% (95% CI: 0.13-0.70), corresponding to 135,706 people (95% CI: 58,224-313,960). CHB prevalence did not significantly differ between men (0.28%) and women (0.32%) (Table 3). Prevalence was significantly higher in persons: i) aged 46-75 years (0.51%) than in those aged 18-45 years (0.10%, *p* < 0.05), ii) in those with the lowest tercile of household monthly income (0.63%) than in those in the 2nd or 3rd terciles (0.14%, *p* < 0.05) and iii) among persons who benefited from specific health insurance for low-income persons (CMU: Complementary Universal Health Insurance or AME: State Medical Insurance for irregular migrants) (1.98%) than those who did not benefit from CMU or AME (0.16%, *p* < 10^− 3^). CHB prevalence was associated with place of birth (*p* < 10^− 3^), reaching 5.81% in persons born in Sub-saharan Africa compared with 0.14% in persons born in mainland France. CHB prevalence was estimated at 3.39% in men who reported at least one male sexual partner in their lifetime (vs. 0.16% in men who did not, *p* < 10-3). CHB prevalence was not significantly higher in persons who reported STI (1.16%) than in those with no history of STI in the previous 12 months (0.30%).

Among those with CHB, an estimated 17.5% (95% CI: 4.9-46.4) were aware of their infection, corresponding to 23,749 (95% CI: 6650-62,967) persons aged 18-75 years in the general population in mainland France.

### HCV and HBV screening history

Among the participants in 2016-HB, 19.2% (95% CI: 18.6-19.9) and 35.6% (95% CI: 34.8-36.5) reported at least one test for HCV and HBV during their lifetime, respectively (Table 2). In multivariate analysis, the likelihood of a HCV and HBV screening history increased with educational level (Table 4). A history of screening for HCV or HBV was more frequently reported by participants living in the Paris urban area or in a city with at least 100,000 inhabitants than by those living in rural areas, and more frequently reported by those benefiting from CMU or AME than those who did not. HCV or HBV screening history was reported more frequently by participants with the following HCV or HBV exposure risks: lifetime drug use, healthcare or prolonged stay in countries with high endemicity for HCV or HBV, blood transfusion before 1992, household or sexual contact with an HBV infected person, lifetime sexual relations with men for male participants, and an STI in the previous 12 months.

**Table 4:** Factors associated with HCV and HBV screening history during lifetime, 2016-Health Barometer, mainland France, 2016 (univariate and multivariate analyses)

Characteristics	HCV (n = 20,029)				HBV (n = 20,029)			
	*wHB* %	aPR	95% CI	p	*wHB* %	aPR	95% CI	p
Gender								
Women	18.6	ref			35.6	ref		
Men	19.9	1.01	0.94-1.09	NS	35.7	1.01	0.96-1.06	NS
Age (in years)	***				***			
15-30	**21.3**	ref			**34.5**	ref		
31-45	**24.4**	1.09	0.99-1.20	NS	**44.2**	**1.23**	**1.16-1.31**	**< 10** ^**−3**^
46-60	**18.6**	0.92	0.84-1.02	NS	**36.5**	**1.22**	**1.14-1.31**	**< 10** ^**− 3**^
61-75	**10.8**	**0.57**	**0.50-0.64**	**< 10** ^**−3**^	**24.8**	0.92	0.85-1.00	NS
Educational level	***				***			
< Secondary school certificate	**15.5**	ref			**30.4**	ref		
Secondary school certificate	**20.2**	**1.22**	**1.11-1.34**	**< 10** ^**−3**^	**37.8**	**1.18**	**1.11-1.25**	**< 10** ^**− 3**^
Higher education qualification	**25.5**	**1.50**	**1.38-1.63**	**< 10** ^**−3**^	**43.9**	**1.28**	**1.21-1.35**	**< 10** ^**−3**^
Household monthly income					***			
1st tercile (low)	18.6				**33.6**	ref		
2nd and 3rd tercile	19.7				**36.9**	**1.09**	**1.03-1.15**	**< 10** ^**−2**^
Place of residence	***				***			
Other regions	**18.1**	ref			**34.5**	ref		
Ile-de-France (Paris region)	**23.9**	**1.12**	**1.02-1.22**	**< 0.05**	**40.3**	1.02	0.96-1.08	NS
Level of urbanization	***				***			
Rural	**15.6**	ref			**31.5**	ref		
< 99,999 inhabitants	**17.7**	1.09	0.99-1.21	NS	**33.6**	1.05	0.99-1.12	NS
≥ 100,000 inhabitants or Paris urban area	**22.0**	**1.17**	**1.06-1.30**	**< 10** ^**−2**^	**38.9**	**1.11**	**1.04-1.18**	**< 10** ^**−2**^
Health Insurance coverage for low-income persons^a^	***				***			
No	**18.9**	ref			**35.2**	ref		
Yes	**25.7**	**1.29**	**1.11-1.50**	**< 10** ^**−2**^	**42.7**	**1.21**	**1.09-1.34**	**< 10** ^**−3**^
HBV endemicity in country of birth^c^					***			
Low					**34.7**	ref		
Intermediate					**39.6**	**1.13**	**1.03-1.23**	**< 10** ^**−2**^
High					**50.1**	**1.22**	**1.08-1.37**	**< 10** ^**− 2**^
HCV endemicity in country of birth^c^	***							
Low	**18.8**	ref						
Intermediate or high	**24.9**	1.15	0.98-1.33	NS				
Blood transfusion before 1992	*							
No	**19.1**	ref						
Yes	**22.1**	**1.33**	**1.17-1.52**	**< 10** ^**−3**^				
Healthcare or stays ≥3 months in Africa, Asia or Middle East	***				***			
No	**18.5**	ref			**34.6**	ref		
Yes	**24.6**	**1.16**	**1.17-1.52**	**< 10** ^**−3**^	**43.1**	**1.09**	**1.01-1.17**	**< 0.05**
Drug use during lifetime	***				***			
No	**18.1**	ref			**34.6**	ref		
Yes, intravenous use with or without nasal use	**64.7**	**3.09**	**2.50-3.82**	**< 10** ^**−3**^	**64.8**	**1.68**	**1.38-2.05**	**< 10** ^**− 3**^
Yes, nasal use only	**37.8**	**1.71**	**1.51-1.93**	**< 10** ^**−3**^	**53.1**	**1.36**	**1.25-1.48**	**< 10** ^**−3**^
Tattooing or piercing without single-use materials								
No	16.9				35.7			
Yes	19.3				33.4			
Lived with or sexual intercourse with an HBV infected person					***			
Yes					**34.7**	ref		
No					**60.7**	**1.55**	**1.43-1.67**	**< 10** ^**−3**^
Sex with other men during lifetime	***				***			
No	**18.8**	ref			**35.3**	ref		
Yes	**40.7**	**1.68**	**1.42-1.98**	**< 10** ^**−3**^	**54.3**	**1.31**	**1.16-1.48**	**< 10** ^**− 3**^
Sexually transmitted infection(s) in the previous 12 months^d^	***				***			
No	**19.1**	ref			**35.4**	ref		
Yes	**37.3**	**1.37**	**1.11-1.69**	**< 10** ^**−2**^	**61.9**	**1.33**	**1.14-1.55**	**< 10** ^**−3**^
HBV vaccination					***			
No					**27.0**	ref		
Yes					**45.7**	**1.59**	**1.51-1.67**	**< 10** ^**−3**^

*wHB %* Health Barometer weighted percentage, *aPR* adjusted prevalence ratio, *95% CI* 95% confidence interval, *NS* Not significant

* *P*-value < 0.05, ** *P* value < 0.01, *** *P* value < 0.001 in bivariate analyses using Chi-2 test

^a^Includes Complementary Universal Health Insurance (CMU, which is free insurance for low-income persons) and State Medical Assistance (AME, which is free insurance for low-income irregular migrants)

^b^Countries with a low level of HCV endemicity: Europe, America, Caribbean; countries with an intermediate or high level of endemicity: Africa, Middle-East, Indian subcontinent, Asia, Pacific Islands

^c^Countries with a low level of HBV endemicity: Northern and Western Europe, North America, Pacific Islands; countries with an intermediate level of endemicity: French Overseas administrative areas, Eastern and Southern Europe, North Africa, Middle-East, Indian subcontinent, South America; countries with a high level of endemicity: Sub-Saharan Africa, Asia

^d^Mycosis are excluded

The distributions are significantly different for numbers in bold

Participants aged 61-75 years old were less likely to report HCV screening than those in the 15-30 years old age group, whereas for HBV, those in the 31-45 and 46-60 years old age groups more frequently reported screening than the 15-30 years old age group. A history of HBV testing was more frequent in participants reporting HBV vaccination. All these results remained unchanged when stratifying for sex.

In total, 32.6% (95% CI: 31.7-33.4) of 2016-HB participants reported that they had never been screened for HCV, HBV or HIV during their lifetime, while 85.3% (95% CI: 84.7-85.9) either reported that they had never been screened for any of the viruses or that they had been screened for only one or two viruses. This corresponds to a minimal estimate of 15,380,061 (95% CI: 14,955,458-15,757,485) and a maximal estimate of 40,242,919 (95% CI: 39,959,851-40,525,988) persons aged of 15-75 years in the general population of mainland France that would need to be screened if the proposed “universal combined screening” strategy were implemented.

## Discussion

Implementing an original approach based on a virological study using home-based blood self-sampling which was nested in a large phone-based survey, our work provides new estimates for the public health burden of CHC and CHB in the general adult population living in mainland France: 133,466 persons (95% CI: 56,880-312,626) had CHC, among whom 80.6% (95% CI: 44.2-95.6) were aware of their infection; 135,706 persons (95% CI: 58,224-313,960) had CHB, among whom only 17.5% (95% CI: 4.9-46.4) were aware of their infection. Our work also provides useful data on HCV, HBV and HIV screening history which can guide the ongoing reassessment of current screening strategies. More specifically, approximately one in five and one in three persons reported HCV and HBV screening during their lifetime, respectively. If a universal (i.e., for all adults at least once in their life) combined (i.e. simultaneous) HCV/HBV/HIV screening strategy were implemented, between 33 and 85% of mainland France’s adult population would be concerned (i.e., between 15 and 40 million people).

To date, the only national survey-based prevalence data for HCV and HBV in France have come from a stand-alone survey conducted in 2004 on 14,500 individuals in social security medical centers [5]. The large sample size of the 2016-Health Barometer survey (2016-HB) together with its innovative sampling design [23], provided an excellent opportunity to implement an innovative nested survey (BaroTest) to collect hepatitis prevalence data while optimizing human and financial resources. Like BaroTest, other studies have also used blood self-sampling. However, they mainly focused on populations at high risk of HIV infection (MSM, black Africans) [31, 32] or sick people [33]. We found only one previous study investigating blood self-sampling in the general population, specifically in women participating in the Norwegian Breast Cancer Screening program [34]. To our knowledge, BaroTest is the first survey on HCV, HBV and HIV screening based on home-based blood self-sampling among the general population. A comprehensive system of reminders was put in place to prompt those who had agreed to participate to carry out the home-sampling (Additional file 1: Figure S1) [22]. Of all those initially invited to participate in the survey, almost 40% returned DBS, demonstrating that home-based blood self-sampling may constitute an effective and inexpensive tool in epidemiological studies.

Thanks to this relatively high participation rate and the large sample size in 2016-HB, almost 7,000 DBS (substantially more than the 5,000 expected) were analyzed, allowing us to estimate prevalence for CHC and CHB despite suboptimal power. We estimated the prevalence of CHC at 0.30% (95% CI: 0.13-0.70) and 0.30% (95% CI: 0.13-0.70) for CHB, in the general adult population in mainland France. In 2004, the national prevalence survey provided estimates of 0.53% (95% CI: 0.40–0.70) and 0.65% (95% CI: 0.45–0.93), respectively [5]. Since 2004, estimates for CHC prevalence in the general population in mainland France have been based on models: 0.42% (95% uncertainty interval: 0.33-0.53) for 2011, and 0.3% (95% uncertainty interval (UI): 0.1-0.3) and 0.29% (95% UI: 0.14-0.34) both for 2015 [4, 16, 17]. For CHB, the only estimate since 2004 has also been model based: 0.5% (95% UI: 0.4-0.7) for 2016 [15]. Due to methodological differences, caution is needed when comparing these estimates. In particular, compared with the 2004 prevalence survey based on venous blood sampling, the use of DBS in BaroTest may have led to a slight underestimation of CHC and CHB prevalences due to a possible lack of sensitivity. However, several studies, including meta-analyses, have shown excellent diagnostic accuracy (with both specificity and sensitivity higher than 98%) using DBS compared with venous blood sampling for the detection of anti-HCV antibodies, HBsAg and HCV RNA [24, 35, 36]. Although not significant, the observed decrease in CHC prevalence in the general population in mainland France may be linked to the decrease in the number of people infected by blood transfusion before 1992, the availability of DAAs, and the almost certain reduction in HCV incidence. The latter point is partly based on the very probable decrease in HCV incidence among drug users, suggested by a reduction in the prevalence of anti-HCV antibodies among this population (from 58.2% in 2004 to 43.2% in 2011) [37]. This decrease coincides with the continuous enhancement of harm reduction measures. In addition, with systematic screening of blood donations since 1992, the risk of transfusion-transmitted HCV infection is now extremely low, estimated at 0.03 per million donations in 2014-2016 [38]. With regard to CHB, the prevalence estimate from BaroTest did not significantly differ from previous estimates [5, 15]. Both our CHC and CHB prevalence estimates are in line with recent estimates in western European countries: 0.2- 0.25% for Germany [17, 39], 0.29% for the UK [17] for CHC; 0.3% for Germany [15, 39], 0.7% for the UK, 0.1% for Ireland [15] for CHB.

As 2016-HB focused on infectious diseases and sexual health, we were able to document the main HCV and HBV risk exposure factors. In the univariate analysis, CHC and CHB prevalences were significantly higher in individuals with well-known risk exposure factors, e.g. intravenous (IV) drug use during lifetime for CHC and being born in Sub-Saharan Africa for CHB. This finding strengthens the validity of our results, although multivariate analysis could not be performed due to the low number of individuals testing positive in BaroTest. Furthermore, CHC and CHB were significantly more frequent among people with a low socio-economic status, as previously demonstrated in the 2004 French national prevalence survey [5]. The absence of a significant difference in CHC prevalence between persons with a history of blood transfusion before 1992 and those without such a history may be explained by a lack of power and possibly by a memory bias. This suboptimal power may also contribute to CHB prevalence not being higher in men than in women. Caution is needed when extrapolating our results to the French general population. Indeed, marginalized populations where CHC or CHB prevalence is likely to be higher were either not represented at all because of the eligibility criteria (e.g., non-French speaking migrants), or probably underrepresented (e.g. drug users, homeless people) given that HB-2016 recruitment was carried out by telephone. This probable underestimation is particularly true for IV drug users as although some BaroTest participants reported IV drug use in their lifetime, this may have referred to past activity. Consequently, CHC prevalence among them (12%) is much lower than among active IV drug users recruited in harm reduction centres (30%) [40].

Among individuals testing positive, an estimated 80.6% were aware of their infection for CHC, but only 17.5% for CHB. These estimates are not robust given the very small numbers of persons testing positive. Consequently, the confidence intervals were large. In addition, given the previously described low level of knowledge regarding viral hepatitis among the French general population [41], questions in the survey regarding screening history could not specify any virological marker. This may have led to an overestimation of the proportion of infected individuals aware of their chronic infection, in particular for HCV. Possible participant confusion between the various types of hepatitis may also have impacted estimations of the percentages of people reporting lifetime screening for one disease or the other. Accordingly, careful interpretation of these values is needed, in particular when creating parallels with the proportions of people aware of their infection. Compared with previous estimates using the same methodology, the proportion of people reporting a history of testing is very close for HCV (19.2% in 2016-HB vs. 19.7% in 2010-HB [42]), but markedly differs for HBV (35.6% in 2016-HB vs. 15.2% in 2010-HB [42] and 27.4% in the 2010-KABP survey [41]). However, in multivariate analysis, factors associated with HCV or HBV screening history were consistent with those previously described, in particular regarding age (with screening more frequently reported by the 31-45 years old group and less often by the oldest group), educational level and level of urbanization of their place of residence [41, 42]. One important result is that after adjustment, HCV and HBV screening were significantly more frequent in individuals reporting risk exposure factors (e.g., IV drug use, being born in an endemic country). These groups constitute the target populations for the current French screening strategy. Having said that, the level of screening is insufficient (e.g., 65% for HCV screening among persons reporting IV drug use, 50% for HBV screening among migrants born in HBV highly endemic countries).

If the proposed universal combined HCV/HBV/HIV screening strategy were recommended, a large proportion of the general population in mainland France would be concerned. Indeed, based on declarative data, an estimated 85% of the general population aged 15-75 years have never been tested for all three diseases (33% have never been tested for any of the three). Given the probable poor reliability of self-reported HCV and HBV statuses [43, 44], this estimate most likely does not reflect the true proportion of individuals who have not been tested for all three viruses. However, if the HAS recommends this strategy, its practical implementation by physicians will rely on patients’ self-reported history of HCV/HBV/HIV screening. Therefore, this estimate is essential when implementing cost-effectiveness analyses. Irrespective of the strategy finally chosen, home blood self-sampling could prove to be an additional tool to enhance screening. Detailed data on its acceptability and feasibility in the context of BaroTest will be the subject of a future article.

## Conclusions

The new estimates which this study provides for CHC and CHB prevalence and for the proportion of individuals aware of their infection in the adult general population living in mainland France, highlight the need for continued efforts to achieve WHO targets [3]. The data collected by this study regarding levels of HCV and HBV screening in this population, will be of great use to guide decision-making regarding which new screening strategy to adopt. In addition, updating these data in a few years will provide the basis for analyzing the real-world effectiveness of the strategy finally implemented [19]. Finally, home-based blood self-sampling may constitute a new effective tool for screening and epidemiological studies in the general population.

## Supplementary information

**Additional file 1: Figure S1.** Operational flow chart, BaroTest Study, 2016. The figure is an operational flow-chart describing the main steps of BaroTest study from the participants’ inclusion to the results reporting to participants.

## Data Availability

The datasets of the national sample of 2016-Health Barometer and BaroTest used and/or analysed during the current study are available from the corresponding author on reasonable request. The datasets of the regional subsamples of 2016-Health Barometer are not publicly available because they were funded by the following Regional Health Agencies: Ile-de-France, Bourgogne Franche-Comté, Midi-Pyrénées and Provence-Alpes-Côte d’Azur. They can be requested from these agencies.

## Abbreviations

AME: State medical insurance for irregular migrants
BT: BaroTest
CHB: Chronic hepatitis B
CHC: Chronic hepatitis C
CI: Confidence interval
CMU: Complementary universal health insurance
DAAs: Direct-acting antivirals
DBS: Dried blood spots
GP: General practitioner
HAS: French national authority for health
HB: Health-Barometer
HBsAg: HBs antigen
HBV: Hepatitis B virus
HCV: Hepatitis C virus
IV: Intravenous
MSM: Men who have sex with men
NRC: National reference center
STI: Sexually transmitted infection
WHO: World Health Organization
