# Supplementary material for: HCV and HBV prevalence based on home blood self-sampling and screening history in the general population in 2016: contribution to the new French screening strategy
Source: BMC Infect Dis. 2019 Oct 28;19:896. doi: 10.1186/s12879-019-4493-2 (PMC6819439; doi:10.1186/s12879-019-4493-2)
Supplement: Supplementary file 1 — Additional file 1: Figure S1. Operational flow chart, BaroTest Study, 2016. The figure is an operational flow-chart describing the main steps of BaroTest study from the participants’ inclusion to the results reporting to participants. [file 12879_2019_4493_MOESM1_ESM.docx]

**Additional file 1: Figure S 1.** Operational flow chart, BaroTest Study, 2016.

**Health Barometer questionnaire by phone**

**Not eligible**

**Proposal of a self-sampling kit to eligible respondents:**

- *Aged between 18 and 75 years old*
- *Having a health insurance*
- *Not under supervision or guardianship*

Between 3 September and 14 October 2012, an online communication campaign was launched on sites popular with the target population, via social networks and at festivals in partnership with a music radio station. The aim of this campaign was to raise awareness of the website and encourage young people to visit it. The website hosted a questionnaire that was presented as a self-risk assessment tool. This questionnaire would allow the eligibility criteria to be verified and to gather the sociodemographic and behavioural characteristics of participants.

**Oral agreement to receive the self-sampling kit**

If the kit was lost or in case of problems using it: **sending of a new kit**

**Raison for refusal**

**Refusal**

Those who were not eligible to participate were redirected towards a page that provided information on Chlamydia infection. Those who were eligible and randomised into the experimental arm (see article 1) were offered the opportunity to have a self-screening kit sent to the address of their choice. They were specifically asked to tick a box to say if they wanted to receive the kit or if they refused it. In cases of refusal or non-selection, they were sent to a page that provided information on Chlamydia infection and locations where traditional screening is carried out. If they accepted, this opened up a form to enter their address. This page needed to be validated for the participant to be registered and to generate an identification number associated with a kit number. A page thanking the person was displayed and they were then redirected towards the homepage of the www.info-ist.fr website. An email confirmation was sent to all participants.

*Health Barometer platform and its dispatch logistics platform*

If participant does not return the samples, **reminders** **by phone**

Day +15

Day +22

If participant does not return the samples, **reminder by email** (if available)

Day+8

**Sending procedure**

1) Print stickers with address

2) Deliver using standard postal service

**Registration of participant**

1. Allocation of a random BaroTest number (BaroTest ID)
2. Recording of postal address
3. Sending address to the dispatch logistics platform

Twice per week, a list of all those who had accepted to receive a self-screening kit was sent to the logistics coordination team. The team then printed the postal addresses onto adhesive labels and stuck them to the envelopes that contained the previously assembled male and female kits.

**Receipt of kit by the participant**

1) Blood self-sampling

2) Completion of consent form

An agreement was made with the national postal service (the post office) to ensure widespread distribution across the country, and to provide easy access to the program. The package could not exceed 3 cm so that it was able to fit through regulation sized letter boxes and into all postal drop-off points.

If necessary, people who accepted to receive a kit were sent a reminder 15 days after the order date. A second reminder was scheduled for 25 days after the date on which the kit was ordered.

**Return of kit and written consent form to laboratory by the participant**

1. Use of pre-labelled pre-paid envelope
2. Place in post box

*Participant*

Ct (and Ng) was diagnosed by the CNR using a Cobas® 4800 system (Roche® diagnostics). The CNR directly informed the participant whether the results were positive or negative. Negative results were sent to the participant via their chosen delivery method: by post or by email. Positive results were only sent by post and were accompanied by a letter recommending that the person see their doctor to receive appropriate treatment. The AIDS Helpline number “Sida Info Service” was provided, so that they could obtain the address of somewhere to go for a free consultation. This information was also accessible on the website through a simplified search system based on entering a postcode, which would allow access to the details of organisations that offered free screening close to where the participant lived. A letter addressed to the doctor was also attached, in order to provide them with details of the program and to remind them of the recommendations issued for treating these types of chlamydia and gonococcus infections.

**Registration** by National Reference Centre (NRC) for hepatitis B, C and Delta

1. Checking consent form
2. Checking general practitioner (GP) address
3. Sending 2 spots to NRC for HIV

**Sending BaroTest ID** of returned envelopes twice a week to the Health Barometer platform

**Biological analyses** in both NRC laboratories

1. Punching each blood spot
2. Performing EIA tests*
3. Performing HIV confirmation test / HVC RNA test if HIV/HCV EIA positive**

**At least one test result positive**

1. Sending results to the GP
2. Informing the participant that GP has the results

*Analytical laboratories*

**Test result negative**

1. Sending results to the participant
2. Sending copy to the GP

*Santé publique France*

**Sending of laboratory results to Santé publique France**

Adapted from ([1](#_ENREF_1))

* Third-generation EIA (aHCV Vitros ECi, Ortho-Clinical Diagnostics, Raritan, New Jersey, USA) for total anti-HCV; automated enzyme immunoassay (VIDAS HBsAg Ultra,BioMerieux, France) for HBsAg; BioRad fourth-generation enzyme-linked immunosorbent assay (Genscreen Ultra HIV Ag-Ab combo assay) for both anti-HIV and p24 antigen ([2-4](#_ENREF_2)).

** Real-time polymerase chain reaction–based method, Abbott RealTime HCV assay (Abbott Molecular, Des Plaines, Illinois) for HCV RNA ([3](#_ENREF_3)); Western Blot (HIV Blot 2.2, or MP Diagnostics) for anti-HIV confirmatory test.

**References**

1. Lydie N, Saboni L, Gautier A, Brouard C, Chevaliez S, Barin F, et al. Innovative Approach for Enhancing Testing of HIV, Hepatitis B, and Hepatitis C in the General Population: Protocol for an Acceptability and Feasibility Study (BaroTest 2016). JMIR research protocols 2018;7:e180.

2. Chevaliez S, Pawlotsky JM. New virological tools for screening, diagnosis and monitoring of hepatitis B and C in resource-limited settings. J Hepatol 2018;69:916-926.

3. Soulier A, Poiteau L, Rosa I, Hezode C, Roudot-Thoraval F, Pawlotsky JM, et al. Dried Blood Spots: A Tool to Ensure Broad Access to Hepatitis C Screening, Diagnosis, and Treatment Monitoring. The Journal of infectious diseases 2016;213:1087-1095.

4. Barin F, Plantier JC, Brand D, Brunet S, Moreau A, Liandier B, et al. Human immunodeficiency virus serotyping on dried serum spots as a screening tool for the surveillance of the AIDS epidemic. Journal of medical virology 2006;78 Suppl 1:S13-18.
